# Circulating MicroRNA Profiles in Pregnant South African Women with Different Types of Diabetes Mellitus

**DOI:** 10.3390/ijms26199337

**Published:** 2025-09-24

**Authors:** Matladi Masete, Stephanie Dias, Nompumelelo Malaza, Sumaiya Adam, Hygon Mutavhatsindi, Carmen Valverde-Tercedor, Begoña Vega-Guedes, Ana Maria Wägner, Carmen Pheiffer

**Affiliations:** 1 Biomedical Research and Innovation Platform (BRIP), South African Medical Research Council, Cape Town 7501, South Africa; 2 Department of Obstetrics and Gynecology, School of Medicine, Faculty of Health Sciences, University of Pretoria, Pretoria 0002, South Africa; 3 Department of Medicine, Faculty of Health Sciences, University of Cape Town, Cape Town 7925, South Africa; 4 Institute of Biomedical and Health Research (IUIBS), University of Las Palmas de Gran Canaria, 35016 Las Palmas de Gran Canaria, Spain; 5 Endocrinology and Nutrition Department, Complejo Hospitalario Universitario Insular Materno-Infantil de Canarias, 35016 Las Palmas de Gran Canaria, Spain; 6 Obstetrics and Gynaecology Department, Complejo Hospitalario Universitario Insular Materno-Infantil de Canarias, 35016 Las Palmas de Gran Canaria, Spain; 7 Non-Communicable Diseases Research Unit (NCDRU), South African Medical Research Council, Cape Town 7501, South Africa

**Keywords:** miRNAs, type 1 diabetes, type 2 diabetes, gestational diabetes, pregnancy

## Abstract

Diabetes in pregnancy increases the risk of adverse perinatal outcomes for mother and child, with severity influenced by the type of diabetes and degree of hyperglycemia. This study aimed to identify circulating microRNAs (miRNAs) associated with different types of diabetes in pregnancy. Serum miRNAs were profiled in pregnant South African women with type 1 diabetes (T1DM), type 2 diabetes (T2DM), gestational diabetes (GDM), and normoglycemia using PCR arrays (*n* = 15). Differentially expressed miRNAs were validated in pregnant South African women (*n* = 167), and a separate cohort of Spanish pregnant women with T1DM and T2DM (*n* = 48). PCR arrays showed significant differential expression for miR-19b-3p (↓ 9.8-fold; *p* = 0.033) in GDM, miR-20a-5p (↓ 4.5-fold; *p* = 0.047) in T1DM, and miR-29a-3p (↑ 1.8-fold; *p* = 0.002) in T2DM compared to normoglycemia. Screening in the larger cohort showed lower expression of miR-20a-5p (↓ 2-fold; *p* = 0.013) in GDM and miR-30d-5p (↓ 2.1-fold; *p* = 0.032) in T1DM compared to normoglycemia. Additionally, miR-20a-5p levels were higher in women with T2DM compared to those with GDM (↑ 2.5-fold; *p* = 0.019). Our findings show that miRNA profiles are largely consistent across different types of diabetes in pregnancy, suggesting that hyperglycemia plays a key role in shaping miRNA expressions. Moreover, the identification of several shared gene targets suggests common underlying pathophysiological mechanisms.

## 1. Introduction

Diabetes in pregnancy poses a serious public health concern due to its association with pregnancy complications and adverse outcomes for both mother and child. In 2021, 16.7% of live births (21.1 million) globally were associated with diabetes in pregnancy [1]. Of these, 10.6% were due to pre-existing type 1 diabetes (T1DM) or type 2 diabetes (T2DM), 9.1% due to T1DM or T2DM diagnosed during pregnancy, and 80.3% were due to gestational diabetes mellitus (GDM), a mild form of glucose intolerance that develops during pregnancy. Perinatal complications associated with diabetes in pregnancy include preeclampsia, caesarean section, preterm birth, macrosomia, shoulder dystocia, neonatal hypoglycemia, hyperbilirubinaemia and congenital malformations [2,3]. After pregnancy, both mothers and their babies have an increased risk of cardiometabolic diseases [4,5]. Pregnancies complicated by T1DM and T2DM are associated with a higher risk of adverse perinatal outcomes compared to GDM [6,7], possibly due to longer exposure and a higher degree of preconception and in utero hyperglycemia [8].

MicroRNAs are short, non-coding RNA molecules between 18 and 25 nucleotides in length that modulate gene expression through post-transcriptional mechanisms [9]. MiRNAs regulate diverse biological processes including cell proliferation, differentiation and apoptosis [10], as well as metabolic processes such as glucose homeostasis, insulin signalling, pancreatic beta-cell function, lipid metabolism and inflammation [11]. Furthermore, miRNA expression has been shown to vary during pregnancy, regulating important metabolic and developmental processes [12]. MiRNAs are produced in the nucleus and are subsequently exported to the cytoplasm to exert their regulatory action on genes. In recent years, circulating miRNAs have been recognized as key mediators of cell–cell communication. They are secreted into the extracellular environment and taken up by recipient cells, where they regulate gene expression and influence various biological processes [13]. Circulating miRNAs are stable, maintaining their integrity through freeze–thaw cycles and RNase digestion. They remain intact for up to 48 h at room temperature and can be stored for as long as 14 years at −80 °C [14], making them promising candidates for biomarker development.

Collares et al. compared miRNAs between non-pregnant women and men with pre-existing T1DM and T2DM, and pregnant women with GDM [15]. Their results showed that nine miRNAs (miR-126, miR-1307, miR-142-3p, miR-142-5p, miR-144, miR-199a-5p, miR-27a, miR-29b, and miR-342-3p) were common to T1DM, T2DM and GDM, while 20 miRNAs were unique to T1DM, 14 were unique to T2DM, and 19 were unique to GDM. Lower levels of circulating miR-126-3p have been reported during T2DM and suggested to serve as a biomarker for inflammation and endothelial dysfunction [16]. MiR-30d-5p and miR-27a-3p were shown to be decreased in placenta and plasma during GDM [17] and T2DM [18] compared to normoglycemia, respectively. This exploratory study aimed to profile circulating miRNAs in pregnant South African women with T1DM, T2DM and GDM. In addition, the expression of miRNAs in the serum of pregnant Spanish women with T1DM and T2DM was quantified to determine if miRNA expression patterns were similar across different populations.

## 2. Results

### 2.1. Participant Clinical and Metabolic Characteristics

Participant characteristics according to diabetes type are summarised in Table 1. Data for women with pregestational T2DM and those with T2DM diagnosed for the first-time during pregnancy were combined due to the absence of notable differences in biochemical markers between the groups. Women with T2DM and GDM were older than women with T1DM. As expected, women with T1DM visited the clinic earlier in pregnancy compared to those with GDM. Therefore, the lower BMI observed in the T1DM group may simply reflect differences in gestational age at the time of measurement. Women with GDM weighed more than women with normoglycemia, T1DM and T2DM, and had lower HbA1c levels than women with pregestational diabetes. However, Glycated hemoglobin (HbA1c) levels were higher in women with T1DM compared to T2DM. Similarly, in a population of pregnant women from Spain, HbA1c levels were higher in women with T1DM compared to those with T2DM (Supplementary Table S1). As expected, women with T2DM had higher 0- and 2-h glucose levels compared to GDM and normoglycemia. A higher proportion of women with T2DM and GDM had a history of hypertension during pregnancy compared to those with normoglycemia. Triglyceride and C-peptide levels were higher in women with T2DM and GDM than in women with T1DM or normoglycemia. No other differences were observed between these groups.

### 2.2. MiRNA Expression Profiling

A total of 36 miRNAs were differentially expressed in pregnant women with diabetes compared to women with normoglycemia, using a 1.5-fold change cut-off (Supplementary Figure S2). However, only the differential expression of three miRNAs was statistically significant. The expression of miR-19b-3p was lower in women with GDM (9.8-fold; *p* = 0.033), miR-20a-5p was lower in women with T1DM (4.5-fold; *p* = 0.047), and miR-29a-3p was higher in women with T2DM (1.8-fold; *p* = 0.002) compared to women with normoglycemia (Table 2).

### 2.3. Validation of Differentially Expressed miRNAs

**Figure 1:**
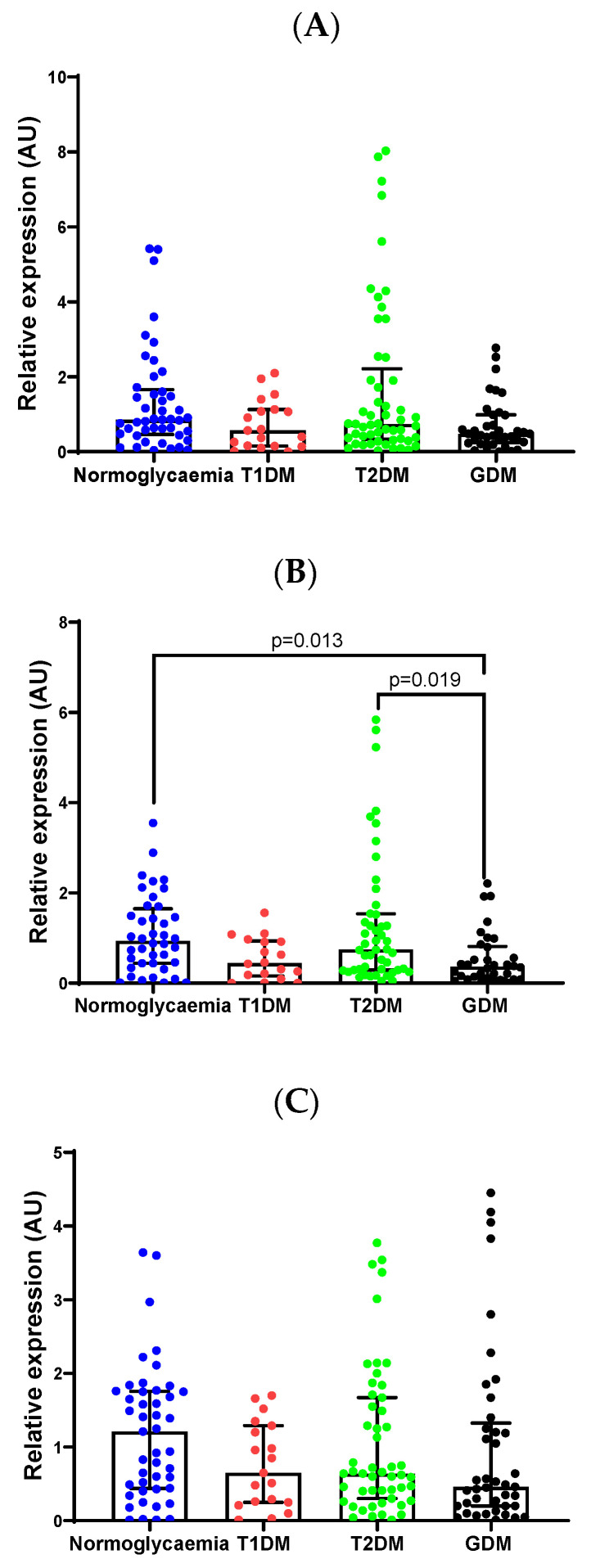

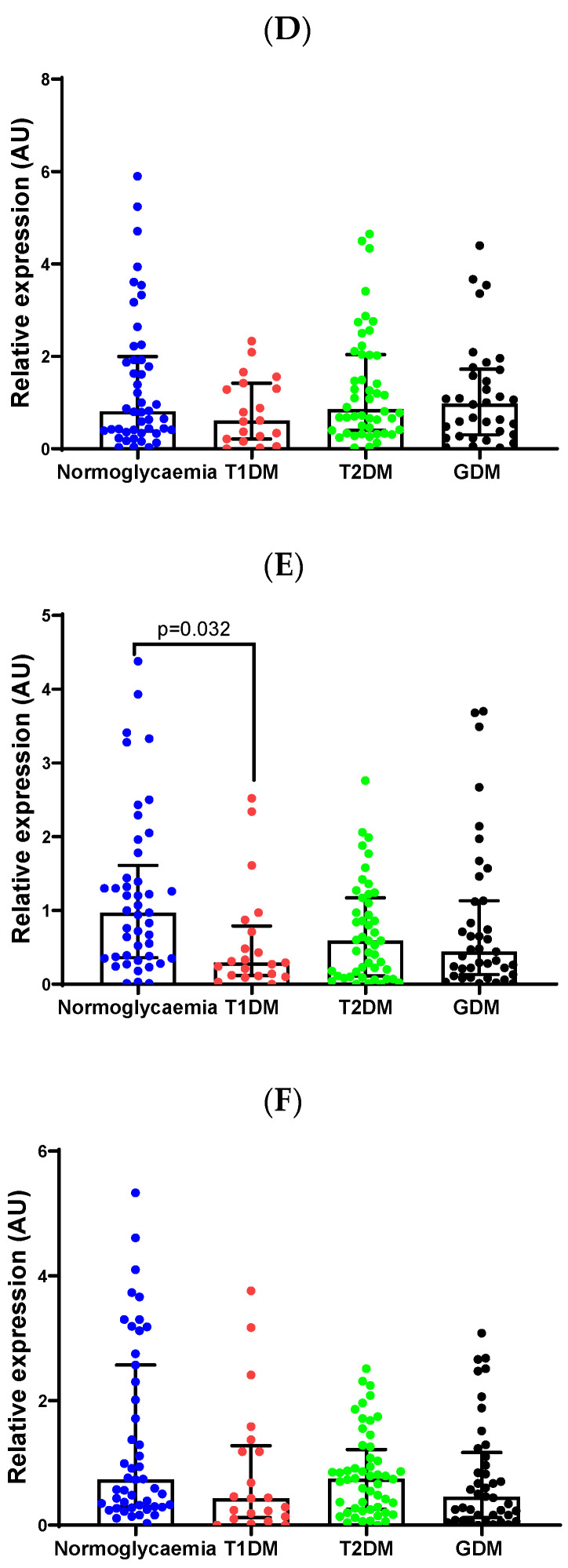
MiR-19b-3p (**A**), miR-20a-5p (**B**), miR-27a-3p (**C**), miR-29a-3p (**D**), miR-30d-5p (**E**) and miR-126-3p (**F**) expression levels were quantified in the serum of pregnant South African women with normoglycemia (*n* = 47), T1DM (*n* = 23), T2DM (*n* = 54) and GDM (*n* = 43). MiRNA relative expression was calculated using the 2^−ΔΔCT^ method. Data are presented as the median and interquartile range (25th and 75th percentiles). Abbreviations: GDM, gestational diabetes mellitus; T1DM, type 1 diabetes mellitus; T2DM, type 2 diabetes mellitus.

To confirm the differential expression of miR-19b-3p, miR-20a-5p and miR-29a-3p identified in PCR arrays and profile candidate miRNAs, miR-27a-3p, miR-30d-5p and miR-126-3p, which were selected based on relevance to diabetes from the literature [16,17,18], qRT-PCR with individual miRCURY LNA PCR assays was performed in a larger sample of pregnant women with diabetes. Validation confirmed lower levels of miR-20a-5p in women with GDM compared to normoglycemia (2.0-fold; *p* = 0.013) and T2DM (2.5-fold; *p* = 0.019) (Figure 1). MiR-30d-5p levels were decreased in women with T1DM (2.1-fold; *p* = 0.032) compared to normoglycemia. However, these results did not withstand Bonferroni correction (Supplementary Table S3). Additionally, no differences in miRNA expression were observed between Spanish women with T1DM and those with T2DM (Supplementary Figure S3).

### 2.4. Association Between Clinical and Metabolic Parameters

We explored the correlation of miR-20a-5p and miR-30d-5p with clinical and metabolic parameters (Age, 0- and 2-h OGTT glucose concentrations, HbA1c, BMI, Gestational age at recruitment, Triglycerides and C-peptide). MiRNAs correlated with each other (rho:0.743; *p* < 0.001). MiR-20a-5p was negatively correlated with triglycerides (rho: −0.176; *p* = 0.025) and C-peptide levels (rho: −0.178; *p* = 0.023). MiR-30d-5p showed a negative correlation with C-peptide levels (rho: −0.218; *p* = 0.005), with a trend towards significance observed with triglycerides (rho: −0.140; *p* = 0.076) (Supplementary Table S2).

### 2.5. Evaluation of miR-20a-5p and miR-30d-5p Discriminatory Ability to Predict GDM and T1DM

**Figure 2:**
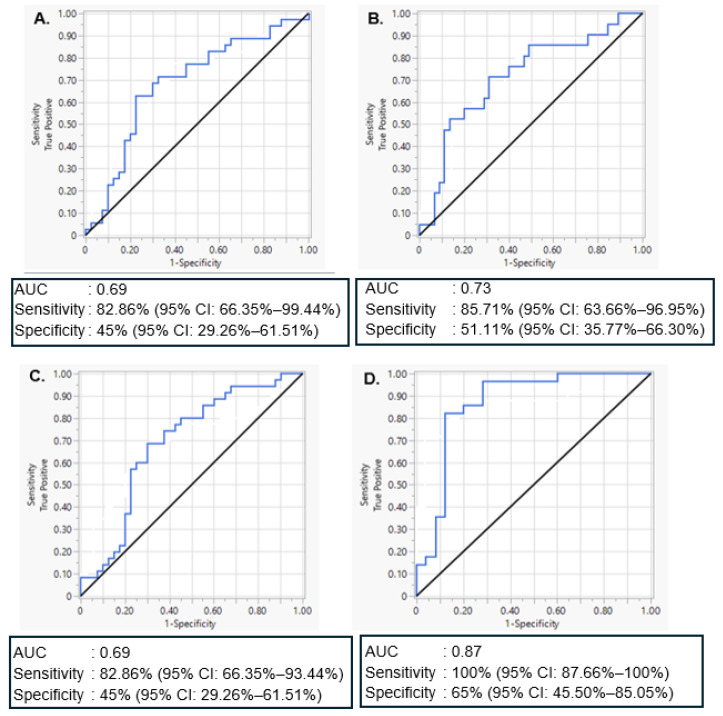
Receiver operating characteristic (ROC) curve showing the accuracy of miRNA biomarkers in distinguishing GDM or T1DM from normoglycaemic controls. (**A**) miR-20a-5p only for discriminating GDM from normoglycemia, (**B**) miR-30d-5p for discriminating T1DM from normoglycemia, (**C**) a combined model of miR-20a-5p and miR-30d-5p for discriminating GDM from normoglycemia, and (**D**) a combined model of miR-20a-5p, miR-30d-5p age, gestational age and BMI for discriminating GDM from normoglycemia. Abbreviations: GDM, gestational diabetes mellitus; T1DM, type 1 diabetes mellitus; AUC, Area under the curve.

Univariate and multivariate discriminant ROC curve analyses were conducted to assess the ability of significantly differentially expressed miRNAs, miR-20a-5p and miR-30d-5p, to distinguish women with GDM or T1DM from women with normoglycemia, respectively. For miR-20a-5p, the analysis demonstrated moderate diagnostic performance, with an AUC of 0.69. Based on Youden’s Index and clinical priorities favouring sensitivity, the optimal threshold yielded a sensitivity of 82.9% (95% CI: 66.4–93.4%) and specificity of 45.0% (95% CI: 29.3–61.5%) (Figure 2A), supporting its potential use as a screening biomarker to detect most GDM cases despite a higher false positive rate. For MiR-30d-5p, discrimination between women with T1DM and normoglycemia was slightly better, with an AUC of 0.73, sensitivity of 85.7% (95% CI: 63.7–97.0%), and specificity of 51.1% (95% CI: 35.8–66.3%) (Figure 2B), indicating stronger individual classification performance in this subgroup. A multivariate model combining miR-20a-5p and miR-30d-5p to discriminate between women with GDM and normoglycemia produced an AUC of 0.69, with a sensitivity of 82.9% (95% CI: 66.4–93.4%) and specificity of 45.0% (95% CI: 29.3–61.5%) (Figure 2C), retaining high sensitivity while modestly improving specificity compared to miR-20a-5p alone. Lastly, a combined model including miR-20a-5p, miR-30d-5p, maternal age, gestational age, and BMI demonstrated the strongest overall performance, with an AUC of 0.87, sensitivity of 100% (95% CI: 87.7–100%) and specificity of 68.0% (95% CI: 46.5–85.1%) (Figure 2D). This model substantially improved classification accuracy, highlighting the additive value of clinical parameters in enhancing miRNA-based discrimination of women with GDM from normoglycemia.

### 2.6. MiRNA Gene Targets and Their Enriched Biological Pathways

Target prediction and pathway analysis were conducted for significantly differentially expressed miRNAs, miR-20a-5p and miR-30d-5p. A total of 46 KEGG pathways and 511 mRNA gene targets were shared between miR-20a-5p and miR-30d-5p. The most commonly enriched pathways included cellular processes such as Tumour Protein P53 (p53) and Wingless-related integration site (Wnt) signalling pathways as shown in Table 3.

## 3. Discussion

Circulating miRNAs have emerged as promising biomarkers associated with diabetes in pregnancy [19]. Accumulating studies have explored the potential of circulating miRNAs as biomarkers for GDM [20], with limited studies investigating T1DM and T2DM during pregnancy [21,22,23]. This study is the first to profile serum miRNA expression in South African women with different types of diabetes during pregnancy and to compare these findings with Spanish pregnant women with T1DM and T2DM. We found that overall miRNA profiles were largely similar across diabetes types, with small but statistically significant quantitative differences. Notably, two miRNAs, namely miR-20a-5p and miR-30d-5p were downregulated in GDM and T1DM, respectively, compared to normoglycemia, highlighting potential biomarkers for diabetes in pregnancy across diverse populations.

Collars et al. [15] have similarly reported that miRNA profiles in individuals with T1DM, T2DM and GDM are generally similar, albeit that participants with T1DM and T2DM were not pregnant and included males [15]. While T1DM, T2DM and GDM have distinct pathophysiological mechanisms, they share similarities in dysregulated metabolic pathways such as glucose metabolism, lipid metabolism, and insulin signaling [24,25]. T1DM is an autoimmune disease characterized by the progressive destruction of pancreatic β-cells, and insulin resistance can develop as the condition progresses [26], while T2DM manifests as chronic insulin resistance which may lead to failure of pancreatic β-cell function over time. Studies have reported that GDM is strongly associated with T2DM and in rare cases with T1DM [27]. Similar aetiologies such as insulin resistance, inflammation and lipid dysregulation [28], as well as common genetic and environmental factors have been reported for T2DM and GDM [29]. Furthermore, women with GDM have a 10-fold increased risk of developing T2DM later in life [30], which supports the notion of a shared aetiology. GDM and T1DM share some risk variants, and a small percentage of women with GDM have tested positive for T1DM autoimmune markers [31]. Differences in the expression of lipid and glucose metabolism genes have been demonstrated in the placenta of mothers with T1DM and GDM [32]. Unfortunately, our study did not assess placental gene expression in mothers with either T1DM, T2DM and GDM. Overall, our findings suggest that hyperglycemia, irrespective of the type of diabetes, may be the primary driver of miRNA patterns in pregnant women with diabetes.

Although miRNA expression patterns were generally similar across maternal groups, quantitative differences were noted. The expression of miR-20a-5p was significantly lower in pregnant women with GDM, consistent with previous findings consisting of 28 women with GDM and 53 women with normoglycemia in a South African population [20]. In contrast, two studies conducted in Chinese women with GDM reported higher expression of miR-20a-5p compared to normoglycemia [33,34]. Both studies analysed plasma miRNAs; however, Zhu et al. [33] conducted qPCR using SYBR Premix Ex Taq II polymerase and relative expression was normalised to endogenous control miR-221, while Cao et al. [34] measured miRNA expression using TaqMan miRNA assays and U6 as an internal control, and relative expression was normalised using *Caenorhabiditis elegans* (*C. elegans*) miRNAs (cel-miR-39, cel-miR-54, and cel-miR-238) as external references. Discrepancies between studies could be due to several factors including differences in sample source, measurement platform and methods used, normalisation (exogenous or endogenous) controls and population differences. Moreover, other differences between studies include, sample size, sample collection and storage, and population factors such as age, ethnicity, BMI and lifestyle factors such as diet and smoking [35,36,37,38]. Additionally, we observed lower expressions of miR-19b-3p during GDM when compared to normoglycemia; however, the difference was not statistically significant. Again, this finding is consistent with Pheiffer et al. [20], who similarly reported a decrease in the expression of miR-19b-3p in women with GDM compared to those with normoglycemia, although not significant [20]. MiR-20a-5p and miR-19b-3p are part of the miR-17-92 cluster [39], which is involved in islet β-cell differentiation and development, insulin resistance and pregnancy abnormalities [19,33,40]. A study by Valverde et al. [23] reported lower expression of miR-20a-5p and miR-19a-3p in HTR-8/SVneo trophoblast cells cultured in T1DM-like conditions compared to cells cultured in normoglycemia [23]. The observed association of miR-20a-5p and miR-19a-3p with both GDM and T1DM further supports our suggestion that hyperglycemia plays a key role in shaping miRNA expression. Lower expression levels of miR-30d-5p were observed in women with T1DM and GDM when compared to normoglycemia; however, statistical significance was only observed for women with T1DM. These findings agree with Zhang et al. [17] who similarly reported lower expression of miR-30d-5p in placental tissue of women with GDM compared to normoglycemia [17]. MiR-30d-5p regulates genes responsible for insulin synthesis and secretion in pancreatic β-cells [41,42] and immune cell development and responses [43]. Lower levels of miR-20a-5p and miR-30d-5p were correlated with higher triglyceride and C-peptide levels, suggesting their involvement in lipid metabolism and insulin secretion [44].

ROC curve analyses demonstrated the potential of selected miRNAs to distinguish women with GDM and T1DM from women with normoglycemia. MiR-20a-5p showed moderate diagnostic performance for women with GDM, with high sensitivity but limited specificity, supporting its use as a screening biomarker where false positives can be resolved by confirmatory testing. MiR-30d better distinguished women with T1DM, achieving high sensitivity and improved specificity. A combined model of miR-20a-5p and miR-30d-5p for women with GDM maintained high sensitivity with modestly improved specificity. Importantly, integrating clinical variables such as maternal age, gestational age, and BMI with miR-20a-5p and miR-30d-5p substantially improved classification performance, yielding an AUC of 0.87 with perfect sensitivity and improved specificity. These findings highlight the promise of combining molecular and clinical markers to enhance early detection of dysglycemia in pregnancy. Given the roles of miR-20a-5p and miR-30d-5p in immune regulation [43], insulin signalling [40], and metabolic inflammation [45,46], their differential expression may reflect disease-specific processes, reinforcing their value in sensitivity-focused diagnostic models. Enrichment pathway target prediction of the significant miRNAs, miR-20a-5p and miR-30d-5p, identified p53 and Wnt signaling pathways that play a role in insulin resistance, impaired glucose regulation and inflammation associated with the increased risk of diabetes [47,48,49].

To the best of our knowledge, this study is the first to comprehensively compare miRNA expression across different types of diabetes in pregnancy. However, our study has several limitations to consider. Due to changes in miRNA extraction kits by the manufacturer, two different exogenous controls were used during miRNA extraction, which may have contributed to variability in the observed miRNA expression levels [50,51,52]. However, we believe this did not introduce significant bias, as both extraction protocols included a representation of the various types of diabetes in pregnancy. Furthermore, miRNAs are influenced by factors including gestational age and environmental factors, such as diet, physical activity, smoking and alcohol consumption, which were not accounted for in this study [53]. The variability in gestational age at the time of blood collection is another limitation of the study; however, it reflects the pragmatic conditions under which the study was conducted. Women with pregestational diabetes tend to present to the clinic earlier in pregnancy [54]. Unfortunately, information on the onset and duration of T2DM was not available, preventing us from assessing the impact of diabetes duration on miRNA expression. Placental tissue is an ideal source for studying disease mechanisms during pregnancy; therefore, the use of serum alone presents a limitation. However, studies suggest that placental miRNAs may be reflected in circulation, which supports our use of serum [55,56]. Blood samples were not collected in a fasting state, which may influence circulating biomarkers such as glucose and lipids. However, current evidence suggests that fasting status has minimal impact on circulating miRNA stability and quantification [57]. Due to the cross-sectional study design, we could not determine whether the miRNA expression changes observed in this study were a cause or consequence of diabetes, and thus future longitudinal studies are warranted to investigate this. In addition, the South African and Spanish cohorts differed in several aspects, including study design, sample collection and participant selection criteria. Notably, the cohorts were not matched at baseline for variables such as age, gestational age and BMI. As a result, comparisons across groups should be interpreted with caution, recognizing the potential for unmeasured confounding and heterogeneity. The findings should be viewed as exploratory and hypothesis-generating, aimed at identifying potential miRNA biomarkers across diverse populations.

## 4. Materials and Methods

### 4.1. Study Population

South African participants were recruited at Steve Biko Academic Hospital in Pretoria, Gauteng, between 2017 and 2023. For this cross-sectional study design, women with diabetes and normoglycemia were recruited at the diabetes antenatal clinic and the general high risk antenatal clinic, respectively. High risk individuals refer to women with obesity, history of hypertension, history of medical conditions such as GDM, and history of adverse pregnancy outcomes. All women provided written informed consent prior to enrolment, and the study was approved by the Human Research Ethics Committee, University of Pretoria (743/2020). The study enrolled a total of 244 participants, of whom only 167 samples [normoglycemia (*n* = 47), T1DM (*n* = 23), T2DM (*n* = 54) and GDM (*n* = 43)] at 7–28 weeks of gestation were selected for miRNAs analysis (Supplementary Figure S1). The eligibility criteria included (1) age between 18 and 42 years, (2) <28 weeks gestation, (3) black African ethnicity, (4) human immunodeficiency virus (HIV) negative and (5) singleton pregnancy. Diabetes in pregnancy was categorised as pregestational T1DM or T2DM if diagnosed prior to pregnancy based on medical records or medication. Women with T2DM, GDM and normoglycemia were diagnosed using the 2-h 75 g oral glucose tolerance test (OGTT) according to the International Association of the Diabetes and Pregnancy Study Group (IADPSG) [58] and World Health Organization (WHO) [59] as shown in Table 4. Bodyweight and height were measured using standard procedures and body mass index (BMI) was calculated as weight (kg)/height squared (m^2^). At the first visit, under non-fasting conditions, whole blood was collected in ethylenediaminetetraacetic acid (EDTA) tubes for glycated haemoglobin (HbA1c) measurement (National Health Laboratory Services, Pretoria, South Africa) and in serum separator tubes (SST) for serum isolation. SSTs were stored at room temperature for approximately 2 h, where after tubes were centrifuged at 4000 rpm for 15 min at 4 °C (OHAUS Frontier™ Multi FC5706, Parsippany, NJ, USA) to separate blood cells from serum. Serum samples were aliquoted and stored at −80 °C until use for up to 2 years.

Spanish participants were pregnant women with pregestational T1DM (*n* = 26) and T2DM (*n* = 22) who delivered at Complejo Hospitalario Universitario Insular Materno Infantil de Gran Canaria (CHUIMI) between 2012 and 2016. The study was approved by the Ethics Committee CEI/CEIM Hospital Universitario de Gran Canaria Dr. Negrín, CEIm (553/2014). All participants provided written informed consent. The exclusion criteria were women with multiple pregnancies, preterm delivery, cardiovascular diseases and GDM in the absence of acute diseases (such as influenza, COVID-19, etc.) any chronic or systemic diseases or major fetal anomalies. During pregnancy, fasting blood samples were obtained in each trimester as part of the clinical routine. HbA1c was quantified by high-performance liquid chromatography (standardized against the Diabetes Control and Complications Trial). In the third trimester, serum samples were collected, aliquoted and stored at −80 °C until use.

### 4.2. Biochemical Parameters

C-peptide levels were measured using enzyme-linked immunosorbent assay kits (Merck, Darmstadt, Germany), while the triglyceride quantification colorimetric/fluorometric kit was used to measure serum triglyceride concentrations (Merck, Darmstadt, Germany), according to the manufacturer’s instructions.

### 4.3. MiRNA Isolation

MiRNA-enriched total RNA was isolated from 200 µL of serum using the miRNeasy Serum/Plasma kit (Qiagen, Hilden, Germany), according to the manufacturer’s instructions. *C. elegans* miR-39 or UniSp2 were added as exogenous spike-in-controls to control for technical variation during RNA extraction. RNA quantity was assessed using the NanoDrop™ One/OneC Microvolume UV-Vis spectrophotometer (Nanodrop Technologies, Wilmington, DE, USA) according to the manufacturer’s instructions.

### 4.4. Human Serum/Plasma miScript miRNA PCR Arrays

To identify differentially expressed miRNAs, RNA samples (*n* = 15) from women with T1DM (*n* = 4), T2DM (*n* = 4), GDM (*n* = 3) and normoglycemia (*n* = 4) were selected within the South African cohort and subjected to quantitative real-time Polymerase Chain Reaction (qRT-PCR) using Human Serum/Plasma miScript miRNA PCR arrays (Qiagen, Hilden, Germany). MiScript miRNA PCR arrays enable the profiling of 84 miRNAs commonly expressed in serum and plasma (https://geneglobe.qiagen.com/us/product-groups/pathway-focused-miscript-mirna-pcr-arrays (accessed on 8 February 2021)). Samples for PCR array analysis were matched for age, gestational age and BMI as far as possible. For complementary DNA synthesis, 40 ng of RNA was reverse transcribed using the miScript II RT Kit and qRT-PCR was performed using miScript SYBR^®^ Green, according to the manufacturer’s instructions (Qiagen, Hilden, Germany). qRT-PCR was conducted on the Quantstudio 7™ Flex Real-Time PCR System (Applied Biosystems, Waltham, MA, USA), and data were uploaded to the Qiagen GeneGlobe Design and Analysis Hub (https://dataanalysis.qiagen.com/pcr/arrayanalysis.php (accessed on 22 March 2021)) for analysis using a Ct (cycle threshold) cut-off < 35 and a 1.5-fold expression difference.

### 4.5. MiRCURY LNA Individual PCR Assays

To confirm PCR array data, a total of 167 samples from women with normoglycemia (*n* = 47), T1DM (*n* = 23), T2DM (*n* = 54) and GDM (*n* = 43) for the South African cohort, while 48 samples from women with T1DM (*n* = 26) and T2DM (*n* = 22) from the Spanish cohort were selected for validation. Thereafter, 10 ng of RNA samples were reverse transcribed using the miRCURY LNA RT Kit and the UniSp6 spike-in-control according to the manufacturer’s instructions (Qiagen, Hilden, Germany). qRT-PCR was conducted using miRCURY LNATM miRNA PCR Assays on miRNAs selected from PCR array based on significance, miR-19b-3p (YP00204450), miR-20a-5p (YP00204292), miR-29a-3p (YP00204698), and miRNAs selected from literature based on relevance to GDM, miR-27a-3p (YP00206038), miR-30d-5p (YP00206047) and miR-126-3p (YP00204227), and miRCURY LNA SYBR^®^ Green (Qiagen, Hilden, Germany). PCR was conducted on the Quantstudio 7™ Flex Real-Time PCR System (Applied Biosystems, Waltham, MA, USA). Relative miRNA expression was calculated using the comparative 2^−ΔΔCT^ method [25], where ΔΔCT was calculated by subtracting the mean ΔCt value of the normoglycemia group from the mean ΔCt of the target sample (T1DM, T2DM or GDM); ΔCt was calculated by subtracting the Ct of the normalising controls from the Ct of the target. The average of *C. elegans* miR-39 or UniSp2, and UniSp6, were used to normalise miRNA expression. Significant outliers were identified and excluded using the GraphPad outlier calculator (https://www.graphpad.com/quickcalcs/grubbs1/ (accessed on 22 March 2021)). This method performs the Grubbs’ test, which calculates the ratio as the difference between the outlier and the mean, divided by the standard deviation [60].

### 4.6. Bioinformatic Analysis

To explore the functional significance of differentially expressed miRNAs, bioinformatic analysis was conducted to identify messenger RNA (mRNA) gene targets and their enriched biological pathways using DIANA tools mirPath v.3 [61]. The Kyoto Encyclopedia of Genes and Genomes (KEGG) pathway was applied, with a false discovery rate (FDR) correction and Fisher’s exact test (hypergeometric distribution) used for enrichment analysis. The pathway union parameters were employed to merge results, and analyses were restricted to the Tarbase v7.0 [62] database for previous experimentally validated miRNA and mRNA interactions.

### 4.7. Statistics

Graphpad Prism^®^ version 8 (GraphPad Software, La Jolla, CA, USA) was used to conduct statistical analysis. Data are presented as the median and interquartile range (25th and 75th percentiles) for skewed data or as the mean and standard deviation (SD) for normally distributed data. Statistical differences between groups were assessed using one-way Anova or the non-parametric Kruskal–Wallis test and Dunn’s multiple comparisons. To further explore differences between groups, an unpaired Student’s *t*-test or a nonparametric Mann–Whitney test was used, where appropriate. A Chi-square test was applied to determine differences for categorical variables. Spearman’s rank correlation was used to investigate relationships between variables. A *p*-value < 0.05 was considered statistically significant. The Bonferroni correction with a cut-off of α/*n* (where *n* is the number of miRNAs) was used to adjust *p*-values for multiple comparisons [63] (Table S3). Generalized discriminant analysis (GDA) and receiver operating characteristic (ROC) curves were used to assess the ability of individual and combined miRNA biomarkers to distinguish women with GDM or T1D from normoglycemic controls. The area under the curve (AUC), sensitivity, specificity, were determined using JMP^®^ Software, version 18.0 (SAS Institute Inc., Cary, NC, USA). Optimal cut-offs were selected using Youden’s Index [64] and additionally optimized for high sensitivity (≥80%) to minimize missed GDM and T1DM cases.

## 5. Conclusions

This study demonstrates that miRNA profiles are largely similar across different types of diabetes in pregnancy, suggesting that hyperglycemia plays a key role in shaping miRNA expression during pregnancy. We observed decreased expression of miR-20a-5p and miR-30d-5p in women with GDM and T1DM, respectively, compared to normoglycemia, with miR-20a-5p also lower in T2DM compared to GDM. A diagnostic model combining clinical variables, maternal age, gestational age, and BMI with miR-20a-5p and miR-30d-5p significantly improved sensitivity and specificity for distinguishing women with GDM from those with normoglycemia. The identification of shared gene targets further suggests common underlying pathophysiological mechanisms. Future studies are warranted to elucidate the functional roles of these differentially expressed miRNAs and their contributions to adverse outcomes in diabetes-complicated pregnancies.

## Figures and Tables

**Table 1:** Clinical and metabolic characteristics of South African pregnant women.

Variable	Normoglycemia (*n* = 47)	T1DM (*n* = 23)	T2DM (*n* = 54) *	GDM (*n* = 43)
Age (years)	31.0 (27.0–36.0) ^c^	29.0 (27.0–32.0) ^d,e^	35.0 (30.0–37.0) ^d^	36.0 (33.0–37.0) ^c,e^
Gestational age at recruitment (weeks)	22.0 (20.0–25.0) ^a,b^	17.0 (14.0–21.0) ^a,d^	21.0 (17.0–25.0) ^e^	26.0 (24.0–26.0) ^b,d,e^
Body mass index (kg/m^2^)	30.1 (26.1–38.5) ^d^	31.2 (23.6–34.0) ^e^	31.9 (28.8–37.7) ^f^	39.7 (33.7–46.5) ^d,e,f^
Glycated haemoglobin (%)	5.1 (4.9–5.5) ^c,d^	9.2 (7.6–10.7) ^c,e^	7.5 (6.3–8.9) ^d,f^	5.7 (5.3–6.1) ^f,e^
0-h blood glucose OGTT (mmol/L)	4.0 (3.8–4.6) ^d,e^	ND	7.6 (7.0–9.2) ^d,f^	5.5 (5.2–6.0) ^e,f^
2-h blood glucose OGTT (mmol/L)	5.0 (4.3–6.2) ^d,e^	ND	12.9 (10.7) ^d,f^	8.2 (6.5–9.5) ^e,f^
^#^ History of hypertension (%): Yes	4.3% ^d,e^	13.0%	31.5% ^e^	25.5% ^d^
Triglycerides (mmol/L)	1.5 (0.5–2.1) ^a^	0.9 (0–2.5) ^b^	2.1 (0.4–3.9)	2.4 (1.5–3.8) ^a,b^
C-peptide (ng/mL)	1.4 (0–3.2) ^a^	0.5 (0–1.2) ^b,d^	1.8 (0.9–2.7) ^b^	2.2 (1.0–4.3) ^a,d^

Data expressed as the median (25th–75th percentile) and for categorical values a ^#^ Chi-square test was conducted (%). Similar superscripts indicate statistical significance, ^a,b^ *p* < 0.05, ^c,d^ *p* < 0.01, ^d,e,f^ *p* < 0.001. * Includes pregestational T2DM and T2DM diagnosed in pregnancy. OGTT was not conducted in women with pregestational diabetes. Abbreviations: GDM, gestational diabetes mellitus; T1DM, type 1 diabetes mellitus; T2DM, type 2 diabetes mellitus; OGTT, oral glucose tolerance test; ND, not determined.

**Table 2:** MiScript PCR array data.

MiRNA	T1DM		T2DM		GDM	
	Fold Regulation	*p* Value	Fold Regulation	*p* Value	Fold Regulation	*p* Value
miR-19b-3p	↓ 5.3	0.500	↓ 2.0	0.180	**↓ 9.8**	**0.033**
miR-20a-5p	**↓ 4.6**	**0.047**	↓ 1.7	0.520	↓ 9.8	0.110
miR-29a-3p	↑ 2.5	0.180	**↑ 1.9**	**0.002**	↑ 2.9	0.200

Fold-regulation in pregnant women with diabetes compared to normoglycemia. Statistically significant values are shown in bold. Abbreviations: GDM, gestational diabetes mellitus; T1DM, type 1 diabetes mellitus; T2DM, type 2 diabetes mellitus; ↓, downregulation; ↑, upregulation.

**Table 3:** KEGG pathways analysis and miRNA gene targets.

KEGG Pathway	*p*-Value	Genes	MiRNAs
Oocyte meiosis	1.53 × 10^−8^	42	miR-20a-5p
			miR-30d-5p
Pathways in cancer	4.93 × 10^−8^	99	miR-20a-5p
			miR-30d-5p
Ubiquitin mediated proteolysis	3.69 × 10^−7^	49	miR-20a-5p
			miR-30d-5p
Lysine degradation	5.10 × 10^−7^	15	miR-20a-5p
			miR-30d-5p
Protein processing in endoplasmic reticulum	3.18 × 10^−6^	53	miR-20a-5p
			miR-30d-5p
Cell cycle	7.28 × 10^−6^	46	miR-20a-5p
			miR-30d-5p
Hippo signaling pathway	1.14 × 10^−5^	42	miR-20a-5p
			miR-30d-5p
mRNA surveillance pathway	2.00 × 10^−4^	32	miR-20a-5p
			miR-30d-5p
p53 signaling pathway	2.00 × 10^−3^	26	miR-20a-5p
			miR-30d-5p
Wnt signaling pathway	2.00 × 10^−3^	42	miR-20a-5p
			miR-30d-5p
Colorectal cancer	2.00 × 10^−3^	21	miR-20a-5p
			miR-30d-5p
RNA transport	1.00 × 10^−2^	44	miR-20a-5p
			miR-30d-5p

**Table 4:** IADPSG, 2010 and WHO, 2013 diagnostic criteria.

Measurement	GDM	T2DM	Normoglycemia
0-h blood glucose OGTT (mmol/L)	≥5.1	≥7.0	<5.1
1-h Glucose (mmol/L)	≥10	-	<10
2-h blood glucose OGTT (mmol/L)	≥8.5–11	≥11.1	<8.5
Random Glucose (mmol/L)	-	≥11.1	-
HbA1c (%)	-	≥6.5	-

One or more of these values must be met or exceeded for the diagnosis of GDM, T2DM, or normoglycemia as per IADSPG [58] and WHO [59] diagnostic criteria. Abbreviations: GDM, gestational diabetes mellitus; T2DM, type 2 diabetes mellitus; HbA1c, glycated haemoglobin; OGTT, oral glucose tolerance test.

## Data Availability

The datasets presented in this study will be made available upon request to carmen.pheiffer@mrc.ac.za.

## Supplementary Materials

The following supporting information can be downloaded at: https://www.mdpi.com/article/10.3390/ijms26199337/s1.

## Abbreviations

The following abbreviations are used in this manuscript:

AUC	area under the curve
BMI	body mass index
DNA	deoxyribonucleic acid
ELISA	enzyme-linked immunosorbent assay
GDM	gestational diabetes mellitus
HbA1c	glycated haemoglobin
HIV	human immunodeficiency virus
IADPSG	International Association of Diabetes and Pregnancy Study Groups Consensus Panel
MiRNAs	MicroRNAs
OGTT	oral glucose tolerance test
PCR	polymerase chain reaction
P53	tumour protein P53
RNA	ribonucleic acid
ROC	receiver operating characteristic
T1DM	type 1 diabetes mellitus
T2DM	type 2 diabetes mellitus
WHO	World Health Organization
Wnt	Wingless-related integration site
