# Supplementary material for: Circulating MicroRNA Profiles in Pregnant South African Women with Different Types of Diabetes Mellitus
Source: Int J Mol Sci. 2025 Sep 24;26(19):9337. doi: 10.3390/ijms26199337 (PMC12524793; doi:10.3390/ijms26199337)
Supplement: Supplementary file 1 [file ijms-26-09337-s001.zip › ijms-3855744-supplementary.pdf]

1. Supplementary Information

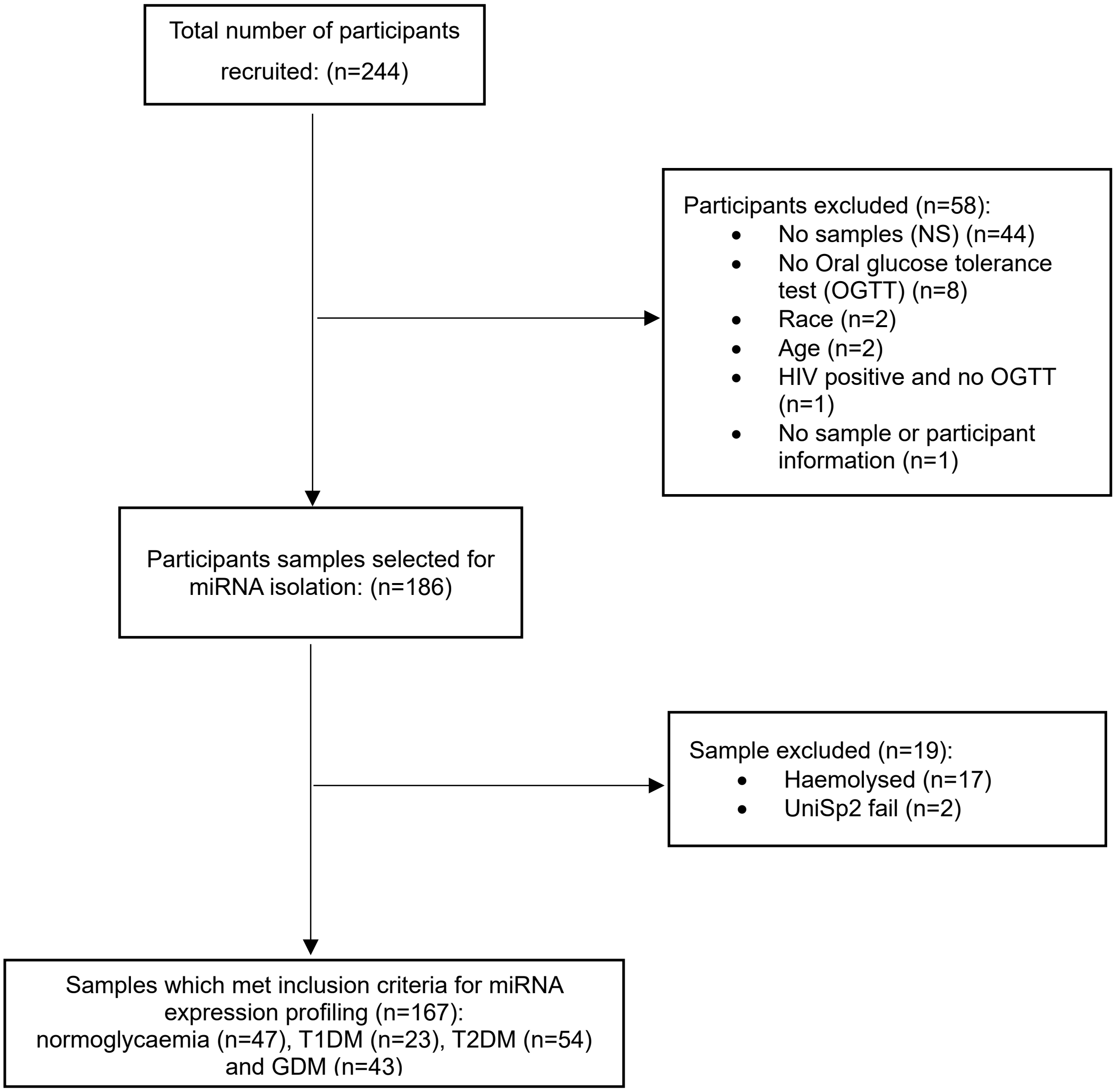

Figure S1. A flow diagram of the study participants sample selection

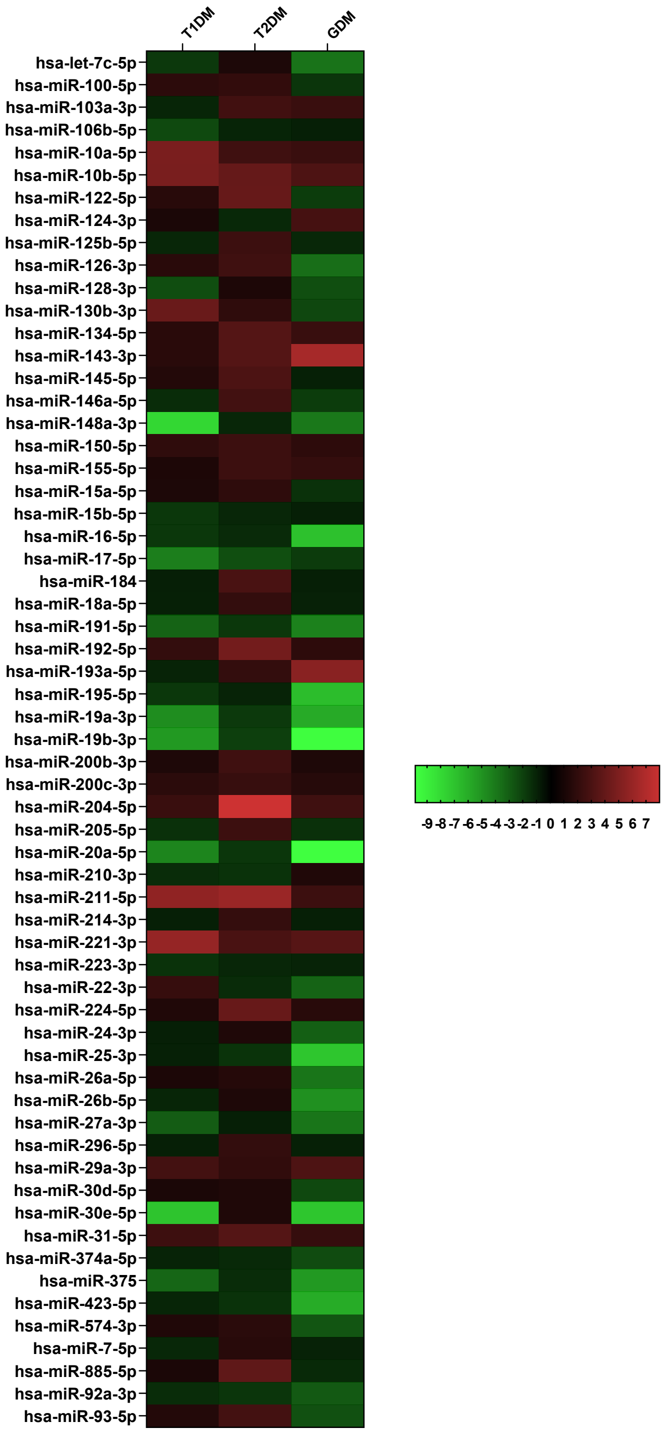

Figure S2. Heat map illustrating differential expression of miRNAs in pregnant women with diabetes compared to normoglycaemia.

MiRNA expression in pregnant women with T1DM (n=4), T2DM (n=4), GDM (n=3) and normoglycaemia (n=4) was assessed using PCR arrays and analysed using Qiagen GeneGlobe Design and Analysis Hub (https://dataanalysis.qiagen.com/pcr/arrayanalysis.php). A cut-off of

1.5-fold was used to assess differential expression. The data are visualised in the heatmap, where the fold regulation compared to normoglycaemia is indicated by the colour intensity. **Abbreviations:** GDM, gestational diabetes mellitus; T1DM, type 1 diabetes mellitus; T2DM, type 2 diabetes mellitus

A. MiR-19b-3p

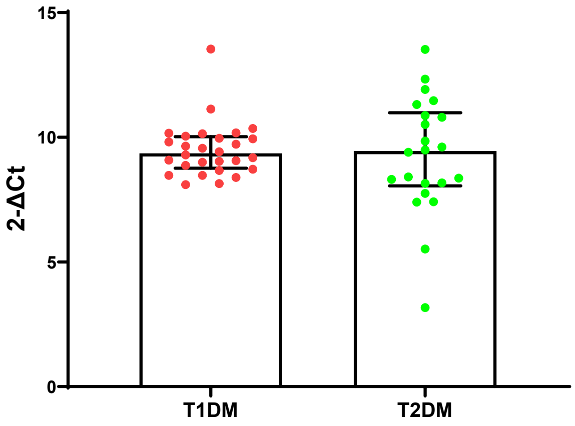

B. MiR-20a-5p

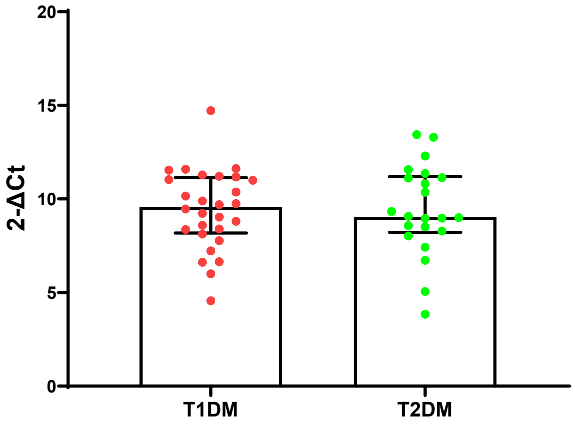

C. MiR-27a-3p

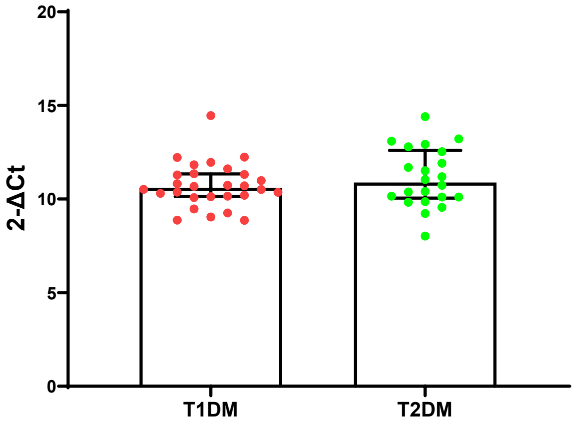

D. MiR-29a-3p

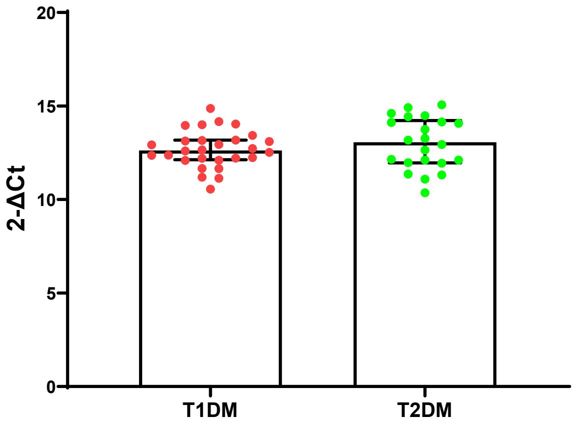

E. MiR-30d-5p

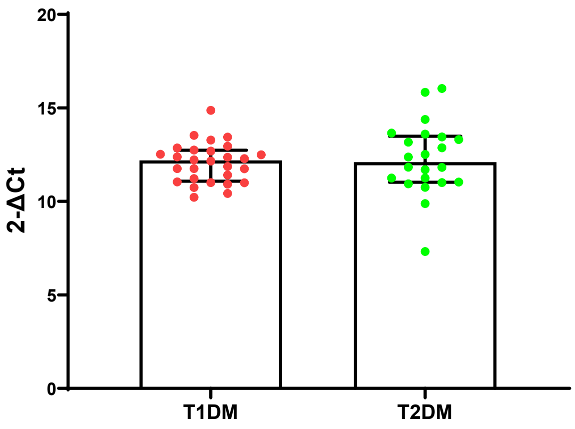

F. MiR-126-3p

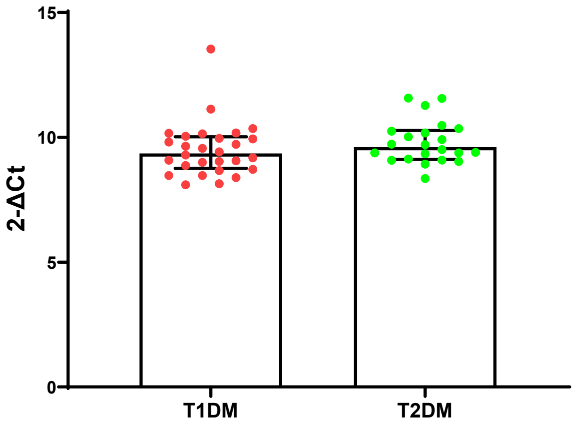

Figure S3. MiR-19b-3p (A), miR-20a-5p (B), miR-27a-3p (C), miR-29a-3p (D), miR-30d-5p (E) and miR-126-3p (F) expression levels were quantified in the serum of pregnant Spanish women with T1DM (n=26) and T2DM (n=22). MiRNA relative expression was calculated using the $2^{-\Delta CT}$ method due to the absence of controls. Data are presented median and interquartile range (25th and 75th percentiles). **Abbreviations:** GDM, gestational diabetes mellitus; T1DM, type 1 diabetes mellitus; T2DM, type 2 diabetes mellitus

Table S1. Clinical characteristics of Spanish pregnant women

Variable	T1DM (26)	T2DM (22)
Age (years)	30.0 ± 5.5	33.0 ± 5.2
Gestational age at delivery (weeks)	38.0 ± 1.0	37.0 ± 7.3
Body mass index (kg/m^2)	26.1 ± 4.7	33.7 ± 6.3
Glycated haemoglobin 1st trimester (%)	6.9 ± 1.0[a]	6.6 ± 1.6[a]
Glycated haemoglobin 2nd trimester (%)	6.0 ± 0.7	5.9 ± 0.9
Glycated haemoglobin 3rd trimester (%)	6.2 ± 0.6	5.9 ± 0.5
Weight at booking (kg)	67.0 ± 12.9	85.5 ± 15.8
Weight in the 3rd trimester (kg)	82.8 ± 14.5	91.7 ± 14.6
Weight gain during pregnancy (kg)	15.5 ± 5.6	8.7 ± 8.0
Proteinuria 1st trimester (gr/24h)	0.1 (0.1-0.1)	0.1 (0.1-0.2)
Proteinuria 2nd trimester (gr/24h)	0.1 (0.1-0.1)	0.2 (0.1-0.3)

Data expressed as the median (25th–75th percentile) for non-parametric distribution or mean ± SD for normally distributed variables. Similar superscripts indicate statistical significance $p<0.05$. Abbreviations: T1DM, type 1 diabetes mellitus; T2DM, type 2 diabetes mellitus.

Table S2. Association between miRNA expression and participant clinical characteristics.

Spearman's rank correlation coefficient (rho)	miR-20a-5p		miR-30d-5p	
	rho	p-value	rho	p-value
Age (years)	-0.040	0.612	0.017	0.826
Gestational age at recruitment (weeks)	-0.099	0.207	-0.109	0.163
Body mass index (kg/m2)	-0.035	0.680	-0.117	0.170
Glycated haemoglobin (%)	-0.056	0.617	-0.074	0.506
0-hour blood glucose OGTT (mmol/L)	-0.079	0.445	-0.121	0.239
2-hour blood glucose OGTT (mmol/L)	-0.110	0.285	-0.118	0.249
Triglycerides (mg/dL)	-0.176	**0.025**	-0.140	0.076
C-peptide (ng/mL)	-0.178	**0.023**	-0.218	**0.005**
miR-20a-5p	1	-	0.743	**2.158E-30**
miR-30d-5p	0.743	**2.158E-30**	1	-

P-values in bold indicate statistical significance.

Abbreviations: OGTT. Oral glucose tolerance test.

Table S3. The Bonferroni correction for multiple comparison.

MiRNA	Diabetes type	P-value (raw)	Bonferroni threshold (α/n, n=6)	Statistically significant (yes or no)
miR-20a-5p	GDM vs normoglycaemia	0.013	0.0083	no
miR-30d-5p	T1DM vs normoglycaemia	0.032	0.0083	no
miR-30d-5p	T2DM vs GDM	0.019	0.0083	no

Abbreviations: T1DM, type 1 diabetes mellitus; GDM, gestational diabetes mellitus; α, significance level (0.05); n, total number of miRNAs.
